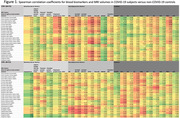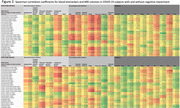# Elevated Blood Biomarkers of Neurodegeneration and Blood Brain Barrier Dysfunction Correlate with Lower MRI Regional Brain Volumes Two Years after COVID‐19

**DOI:** 10.1002/alz.093752

**Published:** 2025-01-09

**Authors:** Jennifer A. Frontera, Yulin Ge, Allal Boutajangout, Wajiha Ahmed, Rebecca A Betensky, Ludovic Debure, Li Jiang, Jon Links, Arjun V. Masurkar, Zhe Sun, Sujata Thawani, Alok Vedvyas, Thomas Wisniewski

**Affiliations:** ^1^ NYU Grossman School of Medicine, New York, NY USA; ^2^ Center for Advanced Imaging Innovation and Research, Radiology, New York University School of Medicine, New York, NY USA; ^3^ NYU Alzheimer’s Disease Research Center, New York, NY USA; ^4^ Alzheimer’s Disease Research Center, New York University Langone Health, New York, NY USA

## Abstract

**Background:**

Cognitive impairment is one of the most frequently reported post‐acute sequelae of COVID‐19, yet the pathophysiology underpinning this symptom remains unknown. We aimed to explore the correlation of blood markers of inflammation, BBB disruption and neurodegeneration with MRI volume measurements in COVID‐19 patients with and without cognitive impairment, and among patients with no prior history of COVID‐19.

**Method:**

We conducted a prospective study of COVID‐19 patients (COV+; laboratory verified SARS‐CoV‐2 infection) and non‐COVID‐19 controls (COV‐; no history of SARS‐CoV‐2 infection and negative SARS‐CoV‐2 nucleocapsid antibody). All patients underwent neuropsychological testing using the Uniform Data Set Version 3 Cognitive Battery. Patients were coded as having cognitive impairment (COG+) based on neuropsychological testing (= 1 abnormal test result), and clinician interview with the patient and informant. 3T MRI (3D T1‐MPRAGE) sequences were utilized for regional volume measurements using MRICloud (Johns Hopkins University) segmentation software. The ratios of regional to total intracranial volume (SPM software) were reported to correct for differences in head size. Fasting plasma biomarkers of neurodegeneration, BBB dysfunction, and inflammation were measured concurrent with MRI testing. Bivariate Spearman correlation coefficients were calculated comparing MRI regional volumetric measures with blood biomarkers among COV+ versus COV‐ subjects with and without cognitive impairment (COG+ versus COG‐). Significance was set at P = 0.001 after Bonferroni correction.

**Result:**

A total of 90 subjects were included (N = 75 COV+ with median 724 days from index SARS‐CoV‐2 infection, N = 15 COV‐; N = 39 COG+, N = 51 COG‐). Among all COV+ subjects (N = 75), neurodegenerative biomarkers (NFL, amyloid‐ß‐40, GFAP, ptau‐181), BBB markers (hepatocyte growth factor) and TNF‐a were significantly negatively correlated with regional MRI volumes, most notably in the limbic and hippocampal regions (Figure 1, bold = P = 0.001); whereas this signature of correlations was not observed in COV‐ subjects (N = 15). Among COV+ subjects with cognitive impairment (COV+COG+, N = 33), significant negative correlations were observed between neurodegenerative biomarkers (NFL, amyloid‐ß‐40, GFAP, ptau‐181) and MRI volumes (particularly frontal, temporal and limbic), whereas COV+COG‐ subjects (N = 42) did not demonstrate this pattern (Figure 2).

**Conclusion:**

Two‐years post‐COVID‐19, signature correlations between neurodegenerative, BBB and cytokine blood biomarkers and MRI regional volumes differ between COVID‐19 subjects versus controls, particularly among those with cognitive impairment.